# Behavioral Changes in Stem-Cell Potency by HepG2-Exhausted Medium

**DOI:** 10.3390/cells9081890

**Published:** 2020-08-12

**Authors:** Francesca Balzano, Giuseppe Garroni, Sara Cruciani, Emanuela Bellu, Silvia Dei Giudici, Annalisa Oggiano, Giampiero Capobianco, Salvatore Dessole, Carlo Ventura, Margherita Maioli

**Affiliations:** 1 Department of Biomedical Sciences, University of Sassari, Viale San Pietro 43/B, 07100 Sassari, Italy; mariafrancesca22@virgilio.it (F.B.); giugarroni21@gmail.com (G.G.); sara.cruciani@outlook.com (S.C.); ema.bellu@hotmail.it (E.B.); 2 Istituto Zooprofilattico Sperimentale della Sardegna, Via Vienna 2, 07100 Sassari, Italy; silvia.deigiudici@izs-sardegna.it (S.D.G.); annalisa.oggiano@izs-sardegna.it (A.O.); 3 Department of Medical, Surgical and experimental Sciences, Gynecologic and Obstetric Clinic, University of Sassari, 07100 Sassari, Italy; capobia@uniss.it (G.C.); dessole@uniss.it (S.D.); 4 National Laboratory of Molecular Biology and Stem Cell Bioengineering of the National Institute of Biostructures and Biosystems (NIBB)-Eldor Lab, at the Innovation Accelerator, CNR, Via Piero Gobetti 101, 40129 Bologna, Italy; ventura.vid@gmail.com; 5 Istituto di Ricerca Genetica e Biomedica, Consiglio Nazionale delle Ricerche (CNR), Monserrato, 09042 Cagliari, Italy; 6 Center for developmental biology and reprogramming-CEDEBIOR, Department of Biomedical Sciences, University of Sassari Viale San Pietro 43/B, 07100 Sassari, Italy

**Keywords:** stem cells, cellular mechanisms, miRNA, exosomes, stemness genes, stem cell differentiation and proliferation

## Abstract

Wharton jelly mesenchymal stem cells (WJ-MSCs) are able to differentiate into different cell lineages upon stimulation. This ability is closely related to the perfect balance between the pluripotency-related genes, which control stem-cell proliferation, and genes able to orchestrate the appearance of a specific phenotype. Here we studied the expression of stemness-related genes, epigenetic regulators (*DNMT1, SIRT1*), miRNAs (*miR-145, miR-148*, and *miR-185*) related to stemness, exosomes, the cell-cycle regulators *p21* (*WAF1/CIP1*) and *p53*, and the senescence-associated genes (*p16, p19*, and *hTERT)*. Cells were cultured in the presence or absence of the human hepatocarcinoma cell line HepG2-exhausted medium, to evaluate changes in stemness, differentiation capability, and senescence sensibility. Our results showed the overexpression of *SIRT1* and reduced levels of *p21* mRNA. Moreover, we observed a downregulation of *DNMT1*, and a simultaneous overexpression of *Oct-4* and *c-Myc*. These findings suggest that WJ-MSCs are more likely to retain a stem phenotype and sometimes to switch to a highly undifferentiable proliferative-like behavior if treated with medium exhausted by human HepG2 cell lines.

## 1. Introduction

Human mesenchymal stem (or stromal) cells are multipotent elements, capable of restoring tissue function after injuries [1]. In particular, stem cells from Wharton jelly (WJ-MSCs) represent a valuable model of these multipotent cells, and can be easily obtained without ethical issues [2,3].

The use of WJ-MSCs is emerging in allogenic transplantation for different kinds of patients, due to their immunomodulatory properties [4,5]. Interestingly, in recent months, intravenous infusion of allogenic WJ-MSCs has been successfully used for the treatment of patients with COVID-19 pneumonia [6,7,8].

The precise combination of signals needed to influence cell fate is highly intricate and not fully understood yet. However, the differentiation of pluripotent stem cells (such as induced pluripotent stem cells (iPSCs) or embryonic stem cells) provides an affordable and streamlined way to reveal the minimum extracellular signals sufficient to specify a given cell type. Moreover, while the influence of specific molecules or conditioned media (secretome) on MSCs cultured in vitro and in animal models is largely, how these cells might react to the paracrine effect of cancer cells in vivo is still unclear [9]. In fact, the behaviors of stem cells and their reprogramming indicate a pathway in which cellular stemness and carcinogenesis seem to be caused by the same sequence of mechanisms. *Sox2*, *c-Myc*, and *Oct4* are the main genes involved in cell reprogramming [10,11]. These factors are indeed used for iPSC generation [12,13]. Several studies have shown that reprogramming factors are closely related to various human cancers, including breast cancer, colorectal cancer, and liver cancer [14,15,16,17]. Expression of transcription factors associated with the maintenance of stemness might be related to a poor prognosis and a significantly shorter total survival [15,16,17]. However, the mechanism underlining the activation of these genes is not clear. *c-Myc* belongs to a family of regulators and proto-oncogenes encoding for essential nuclear transcription factors. *c-Myc* mainly regulates cell growth, proliferation, differentiation, cell cycle, metabolism, survival, and apoptosis, as well as tumorigenesis [18,19,20]. Moreover, it controls tumor cell fate by inducing stemness and blocking cellular senescence and differentiation, and also orchestrating changes in the tumor microenvironment [21,22,23,24,25].

*Oct-4* is a transcription factor [26,27,28,29,30] associated with the pluripotent properties of stem cells, crucial in controlling early stages of mammalian embryogenesis [30,31,32]. In addition, as a key stem-cell marker, *Oct-4* is also involved in lineage specification and in the reprogramming of somatic cells in vitro [33,34]. *Oct-4* is also re-expressed in different types of cancer stem cells, which are clusters of tumor cells at the origin of tumor resistance to chemotherapy and tumor recurrence [34]. Epigenetic changes are likely to play critical roles in both stemness and tumorigenesis [35,36,37,38,39]. Lineage-specific DNA methylation patterns, which are established during embryonic development, are generally faithfully maintained in differentiated adult cells. Within this context, DNA (cytosine-5)-methyltransferase 1 (*DNMT1*) exerts a key role, catalyzing DNA methylation. It is encoded by the *DNMT1* gene [23,24,25,26].

Previous studies have shown that DNA methyltransferase (*DNMT1*) inhibitors and treatment with histone deacetylase (*SIRT1*) modifiers could significantly increase the efficiency of the reprogramming process, and also of carcinogenesis [35].

The contribution of niches to the onset and progression of tumors is not clear. Niches are distinct regions within the tumor microenvironment which maintain the main properties of cancer stem cells (CSCs), preserving their phenotypic capability, protecting them from the environment, and ultimately facilitating their metastatic potential [33,34,35].

In the present study, we evaluated the effects elicited by medium exhausted using the human HepG2 tumor cell line [37,38,39] on the specific cellular and molecular behaviors of WJ-MSCs, in the attempt to reproduce in vitro a modified environment (niche) able to induce a dysregulation in stem-cell proliferation/differentiation. Nevertheless, these special microenvironments, also described in hepatic tissue, consist of different cell types, eliciting a paracrine effect, as well as the extracellular matrix (ECM) scaffold [37,38,39]. Within this context, other authors previously described that hepatic progenitor cells (HPCs), able to differentiate toward different lineages, like multipotent stem cells, may be transformed into cancer stem cells able to lead to hepatocarcinoma tumorigenesis, and that HPC niches or microenvironments are able to ultimately define cell fate [40].

Here, we analyzed the specific role of the cell-cycle arrest/progression compartment by evaluating the expression of the following genes: *p21* (*WAF1/CIP1*), *p19* (*ARF*), *p16* (*INK4A*), *p53*, and *hTERT*. We also evaluated the expression of stemness-related genes *NANOG*, *Oct-4*, *SOX2*, and *c-Myc*, and epigenetic modulator genes *DNMT1*, *SIRT1*, and *GAPDH*.

In a previous paper, we revealed that some microRNAs cooperate with stemness genes to influence WJ-MSCs’ plasticity [41,42,43]. MicroRNAs are post-transcriptional mediators of gene expression and regulation, and also play influential roles in tumorigenesis and cancer metastasis.

Extracellular miR-145 was recently detected in exosomes or microvesicles isolated from body fluids [41,42,43]. In particular, miR-145, together with miR-185 and miR-148, seems to be involved in a range of tumorigenic functions, such as the regulation of cell proliferation, differentiation, apoptosis, and metastasis [41,42,43]. Here, we analyzed the expression of miR-145, miR-185, and miR-148 both in WJ-MSCs and in exosomes of culture media [43,44,45,46].

## 2. Materials and Methods

The study included umbilical cords (*n* = 12) retrieved from healthy full-term women between 25 and 35 years old, recruited according to the following criteria: spontaneous birth, and donors free from drugs, smoking, and diseases. All the experiments were performed twice (in three technical replicates), separately for each of the 12 samples.

### 2.1. WJ-MSC Isolation and Culture

Fresh human umbilical cords (*n* = 12) from both sexes were collected after natural childbirths at the Gynecologic and Obstetric Clinic of the University of Sassari. The patients gave written informed consent according to the approval of this study by the Ethics Committee (Ethical Clearance N. 0021565/2018, 22/03/2018, Commissione Etica CNR). The umbilical cords were collected in phosphate-buffered saline (PBS) supplemented with 200 U/mL penicillin (Euroclone, Milano, Italy), 200 mg/mL streptomycin (Euroclone, Milano, Italy), and 4 mg/mL amphotericin B (Gibco Life Technologies) prior to storage at 4 °C for further WJ-MSC isolation. Tissues were dissected into small pieces and then digested with collagenase type I (2 mg/mL) Sigma at 37 °C for 16–18 h with agitation. After neutralization of the enzyme with 10% fetal bovine serum (FBS) (Life Technologies, Grand Island, NY, USA) and filtering (70 μm cell strainer) (Euroclone, Milano, Italy), samples were centrifuged at 600× *g* for 10 min and cultured in a basic medium (BM), Dulbecco’s modified Eagle’s Medium (DMEM) (Life Technologies Grand Island, NY, USA) supplemented with 10% fetal bovine serum (FBS) (Life Technologies, Grand Island, NY, USA), 200 mM l-glutamine (Euroclone, Milano, Italy), and 200 U/mL penicillin–0.1 mg/mL streptomycin (Euroclone, Milano, Italy), and cultured in T25 flasks at 37 °C with 5% CO_2_ and saturated humidity for 10–14 days [44]. After 48 h of incubation, cultures were washed with PBS and kept in the fresh medium. The culture medium was changed every 3 days. When cells reached 80–90% confluence, they were harvested using 0.25% Trypsin EDTA (Euroclone, Milano, Italy), counted and transferred into new flasks. WJ-MSCs were immunomagnetically sorted for c/kit using a monoclonal anti-c/kit (CD117) antibody (Miltenyi Biotech, Minneapolis, MN, USA) directly conjugated to microbeads (Miltenyi Biotech). The WJ-MSCs used in this study were characterized by flow cytometry as previously described [45,46,47,48].

### 2.2. HepG2

HepG2 cells were cultured as previously described [37,38,39]. Cells secrete many plasma proteins, such as albumin and fibrinogen, acute-phase proteins, alpha 2-macroglobulin, alpha 1-antitrypsin, transferring plasminogen, insulin-like growth factor-binding protein 1, alpha-fetoprotein, and others [37,38,39]. The HepG2 cells were seeded in a basic medium (BM), Dulbecco’s Eagle’s Medium (DMEM) (Life Technologies Grand Island, NY, USA) supplemented with 10% fetal bovine serum (FBS) (Life Technologies, Grand Island, NY, USA), 200 mM l-glutamine (Euroclone, Italy), and 200 U/mL of penicillin – 0.1 mg/mL of streptomycin (Euroclone, Milan, Italy), and cultured in incubator at 37 °C with 5% CO_2_ and saturated humidity for 7 days [37]. When the cells reached 80–90% confluence, the waste medium was collected, after which 1 mL of HepG2 [37,38,39] waste medium was filtered and added to 25 cm^2^ flasks containing WJ-MSCs.

### 2.3. Treatment and Preparation of WJ-MSCs

The WJ-MSCs were then expanded in a modified base medium (BM), Dulbecco’s Eagle’s Medium (DMEM) (Life Technologies Grand Island, NY, USA) integrated with 1 mL of HepG2 waste medium [37,38,39]. The flasks were placed in the culture incubator at 37 °C with 5% CO_2_ and saturated humidity for 7 days. After 7 days of incubation, the cultures were collected using 0.25% of EDTA trypsin (Euroclone, Milan, Italy), counted, and RNA was extracted.

### 2.4. RNA Extraction and Quantitative Polymerase Chain Reaction

After treatment, total RNA was isolated using TRIzol® reagent and quantified by measuring the absorbance at 260/280 nm (NanoDrop 2000, spectrophotometer Thermo Scientific ND8008, Thermo Fisher Scientific, Waltham, MA, USA). Approximately 1 µg of total RNA was reverse-transcribed to cDNA by SuperScript® VILO™ cDNA Synthesis Kit (Life Technologies, Grand Island, NY, USA). Quantitative polymerase chain reactions were performed using a CFX Thermal Cycler (Bio-Rad) in triplicate (Applied Biosystems), incubated under standard qRT-PCR conditions (50 °C for 2 min, 95 °C for 2 min, and then cycled at 95 °C for 15 s, 55–59 °C for 30 s, and 60 °C for 1 min, for 40 cycles), according to the qRT-PCR protocol specified in the Quantitative PCR Master Mix with Power SYBR® Green. For each reaction, 0.1 µM of each primer, and 3 µL cDNA generated from 1 μg of the total RNA template were mixed in 25 µL volumes and added. Target Ct values were normalized to HPRT1 [45,46,47], considered as a reference gene, while the gene levels of stem cells were expressed as fold-change (2−∆∆Ct) relative to the gene levels observed when stem cells reached 80% confluence before starting treatment. Each experiment included a distilled water control. The qRT-PCR analysis was performed for the following set of genes: *NANOG, Oct-4, SOX2, c-Myc, DNMT1, p21 (WAF1 / CIP1), p19 (ARF), p16 (INK4A), p53*, and *hTERT*. All primers used (Invitrogen) are described in Table 1.

### 2.5. RNA Extraction and Quantitative Polymerase Chain Reaction for miRNA

RNA was extracted from cells using Mirvana MIRNA ISO Kit 10-40ISO (Life Technologies) according to the manufacturer’s instructions, with a final elution volume of 15 μL. Three individual miRNAs (hsa-miR-148a-3p, hsa-miR-185-3p, and hsa-miR-145-5p) were selected based on amplification efficiency and previous studies by other authors [49].

### 2.6. Exosome Experiments

Exosomes were isolated from WJ-MSC waste medium using total exosome isolation reagent (Invitrogen). The experiments were performed after 1 and 7 days of culture. Samples were then processed for hsa-miR-148a-3p, hsa-miR-185-3p, and hsa-miR-145-5p extraction and amplification with real-time PCR, as previously described [49].

### 2.7. Quantitative-PCR Analysis

The concentration level of mature miRNAs was tested by quantitative real-time PCR (qPCR) using TaqMan^®^ MicroRNA Reverse Transcription Kit, (Life Technologies) for the reverse transcription. TaqMan^®^ Universal Master Mix II, Life Technologies, was used for the PCR according to the manufacturer’s instructions; 45 amplification cycles were performed. miRNA concentration levels were quantified using the IQ5 BIORAD instrument, (Milan, Italy). The U6snRNA was used for data normalization [50]. Real-time PCR was done in duplicate. The sequences and the identification symbols were retrieved from miRbase and are reported in Table 2.

### 2.8. Gene Expression: Statistical Analysis and Real-Time PCR Data Analysis

The Kruskal–Wallis test was applied to compare the groups (male and female) for each target. The statistical analysis was performed with the SPSS software version 17.0. Reverse transcription followed by polymerase chain reaction (RT-PCR) is the most suitable method for the detection and quantification of mRNA. It provides high sensitivity, good reproducibility, and a wide range quantification. Several mathematical algorithms have been developed to calculate a ratio of expression based on real-time PCR efficiency and the crossing point deviation of an unknown sample against a control. A software tool named REST© (relative expression software) [51] was used, which compares two or more groups and different reference and target genes. The mathematical model used was based on the correction for exact PCR efficiencies and the mean crossing point deviation between sample group(s) and control group(s). Subsequently the expression ratio results of the investigated transcripts were tested for significance using a pairwise fixed reallocation randomization test and plotted using standard error (SE) estimation via a complex Taylor algorithm. Expression variation for each gene was visualized in a box-and-whisker plot. In this study, the relative expression of the mRNAs was analyzed using REST software [51]. The non-parametric bootstrapping test was used to evaluate expression differences between treated and untreated samples.

### 2.9. miRNA: Statistical Analysis and Real-Time PCR Data Analysis

The raw Ct values for each miRNA and U6snRNA were checked for normal distribution. The Kruskal–Wallis test was applied to compare the groups in each target. All the analyses were performed, and graphics generated using SPSS software version 17.0. A software tool named REST^©^ (relative expression software) [51] was used to compare two groups, with a maximum of 16 data points in a sample and 16 in a control group, for reference and up to four target genes. The non-parametric bootstrapping test was used to evaluate concentration differences of miRNAs between male and female samples. Data were analyzed using Statistical Package for the Social Sciences version 13 software (SPSS Inc., Chicago, IL, USA). Krustal–Wallis rank sum and Wilcoxon signed-rank tests were applied to evaluate the distributions of each group variance at different times of observation, assuming *p*-values < 0.05 as statistically significant. WJ-MSCs before and after treatment with HepG2-exhausted medium were compared with untreated cells by statistical analysis. Intrasexual analyzes were also conducted to compare the results between control cells and cells under study. Continuous parametric variables were analyzed using Student’s unpaired *t*-test.

### 2.10. Immunostaining

Treated and control cells were seeded at the concentration of 10,000 cells/well in 8-well Falcon culture slides (BD Falcon, BD Biosciences, Bedford, MA, USA). Once attached, cells were fixed with 4% paraformaldehyde (Sigma Aldrich Chemie GmbH, München, Germany) for 30 min at room temperature. After permeabilization by 0.1% Triton X-100 (Life Technologies, USA)-PBS, cells were washed three times for 5 min each in PBS and incubated with 3% bovine serum albumin (BSA)–0.1% Triton X-100 in PBS (Life Technologies, USA) for 30 min. Cells were then exposed overnight at 4 °C to the primary anti-rabbit anti-GAPDH antibody (Cell Signaling Technology, Danvers, MA, USA) and anti-mouse anti-Sirt1 antibody (Cell Signaling Technology, Danvers, MA, USA). Finally, cells were washed two times in PBS for 5 min each and stained for 1 min at 37 °C in the dark with the fluorescence-conjugated goat anti-rabbit and anti-mouse IgG secondary antibodies (Life Technologies, Carlsbad, CA, USA). Nuclei were labeled with 1 µg/mL 4,6-diamidino-2-phenylindole (DAPI). All microscopy analyses were performed with a confocal microscope (TCS SP5, Leica, Nussloch, Germany).

### 2.11. Alizarin Red Assay

Cells were cultured for 21 days on 24 well tissue culture plates (BD-falcon, BD Biosciences, Bedford, MA, USA), in the presence of StemPro™ Osteogenesis Differentiation Kit (TRT) medium (Life Technologies, USA) or basic growing medium (CTRL). The positive control for osteogenic differentiation (CTRL+) was represented by WJ-MSCs cultured in osteogenic medium. After 21 days, cells were fixed with 10% formalin for 15 min at RT, washed three times in distilled water (ddH_2_O), and then were stained with 2% alizarin red S solution (Santa Cruz Biotechnology, Dallas, Texas, USA) for 20 min at RT. Cells were thoroughly washed several times in ddH_2_O and observed by light microscopy to analyze calcium deposition.

## 3. Results

### 3.1. Gene Expression

Here, we investigated the relative expression levels of the *c-Myc, Oct-4, DNMT1, NANOG*, SIRT1, *p53, SOX2, GAPDH, p21waf1, p19, p16*, and *TERT* genes in WJ-MSCs before and after treatment with HepG2-exhausted medium. The results of the analysis performed with the Normfinder software suggested *HTRP1* as the normalizer (data not shown). We performed a comparison between the expression levels of these genes in WJ-MSCs exposed to HepG2-exhausted medium and untreated WJ-MSC samples used as controls.

**Figure 1:**
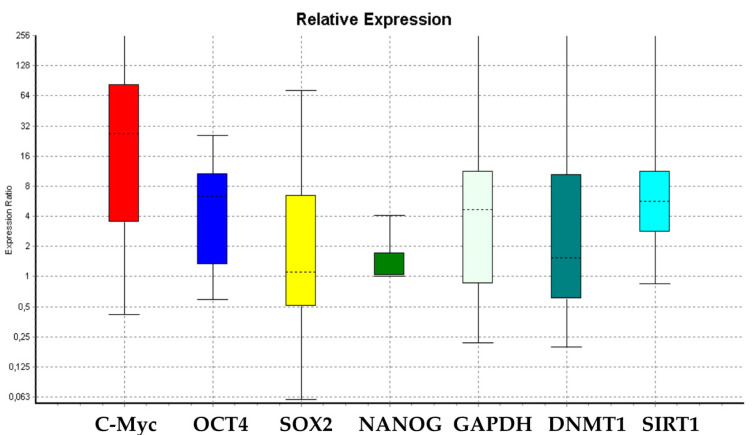
Boxplots representing the expression variation of genes analyzed in this study in the WJ-MSCs after treatment with HepG2-exhausted medium [46] as compared to untreated WJ-MSCs.

**Figure 2:**
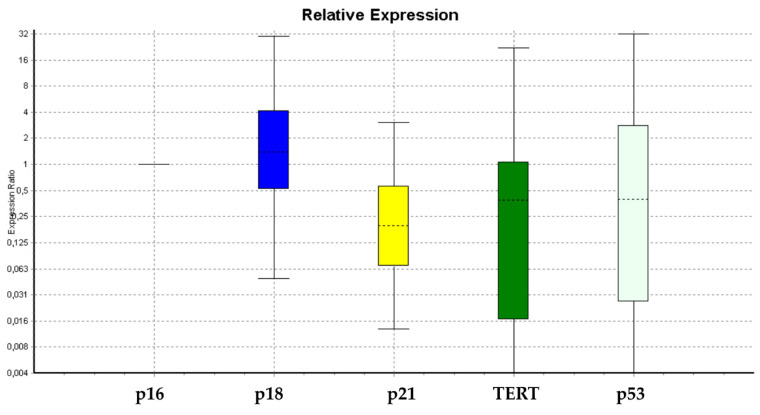
Lower expression of p21 in the treated samples compared to the untreated controls.

Figure 1 and Figure 2 show that there are no significant differences in the expression levels of *SOX2, NANOG, GAPDH, p19, p16, TERT*, and *SOX2* between treated WJ-MSCs as compared to untreated WJ-MSCs. On the other hand, the expression levels of *c-Myc, Oct-4*, and *SIRT1* were significantly increased in treated WJ-MSCs as compared to untreated WJ-MSCs (Figure 1). It is noteworthy that the expression levels of *p21, p53*, and *DNMT1* were decreased in treated WJ-MSCs as compared to untreated WJ-MSCs (Figure 1 and Figure 2). The expression levels of *p19, p16, p53*, and *TERT* did not show significant differences between the two groups of WJ-MSCs, exposed or not to HepG2-exhausted medium (Figure 2).

#### 3.1.1. miRNA

**Figure 3:**
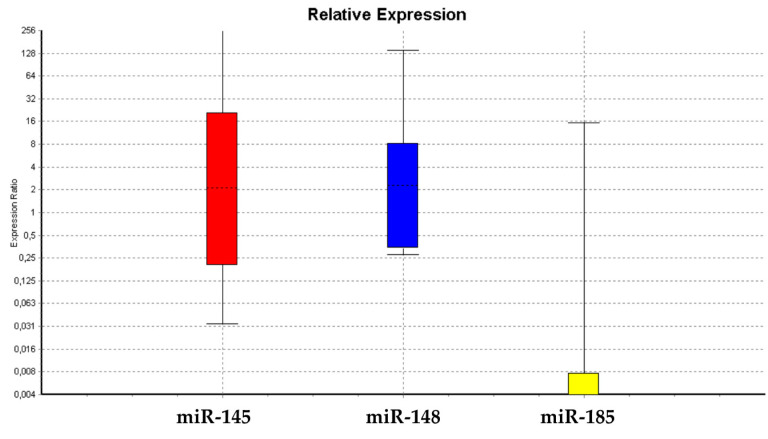
Expression of miRNAs. The graph shows trends in miRNA expression, displaying miR-145-5p, miR148a-3p, and miR-185-3p of WJ-MSCs treated with HepG2-exhausted medium as compared to untreated WJ-MSCs.

Statistical analysis showed that data were not normally distributed. We analyzed three miRNAs—miR-145-5p, miR-148a-3p, and miR-185-3p—using U6snRNA as a normalizer. Statistical analysis supported our decision to use U6snRNA as a normalizer for PCR analysis in real time, due to its greater stability between groups of samples [49]. The Kruskal–Wallis test showed no significant differences in WJ-MSCs before and after treatment with HepG2-exhausted medium [37,38,39], for the expression of miR-145 and miR-148 (Figure 3). On the other hand, the relative expression of miR-185-3p was significantly decreased in treated cells as compared to untreated cells (Figure 3).

#### 3.1.2. Exosomes

**Figure 4:**
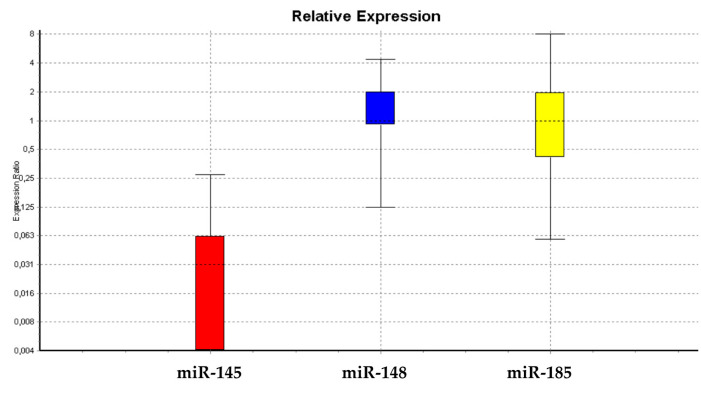
Expression of miRNA exosomes in the HepG2-exhausted medium after one day of treatment. The graph shows the trend in miRNA expression for miR-145-5p, miR148a-3p, and miR-185-3p.

**Figure 5:**
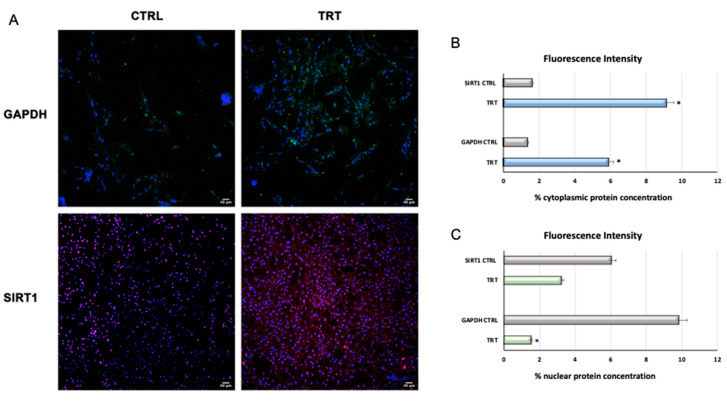
(**A**) Analysis of GAPDH and SIRT1 localization in control and treated WJ-MSCs. Immunohistochemical analysis of the expression of GAPDH and SIRT1 was assessed in control (CTRL) and treated (TRT) cells. Nuclei are labeled with 4,6-diamidino-2-phenylindole (DAPI, blue). Scale bars: 40 µm. (**B**,**C**) The fluorescence intensity was calculated using ImageJ as % of cytoplasmic and nuclear protein concentration, in control and treated cells. Data are expressed as mean ± SD and are representative of n different experiments (*p* < 0.05). Averages were calculated from three technical replicates. Results are representative of two separate experiments.

Figure 4 show that miR-145 was less expressed in exosomes isolated from exhausted-medium-treated WJ-MSCS as compared to untreated cells soon after one day of treatment. The same miRNA could not be detected in the medium of treated WJ-MSCs after a week, while being still present in untreated control WJ-MSCs. The expression levels of miR-148 and miR-185 detected in the exosomes did not show any significant differences between treated and untreated WJ-MSCs. Our results showed that miR-145 was not expressed in exosomes isolated from HepG2-exhausted medium. (Figure 4 and Figure 5).

#### 3.1.3. Immunostaining

The results showed the expression and localization of GAPDH and SIRT1 in control and treated cells. GAPDH was detected both in the cytosol and in the nuclei of both cells. Nevertheless, as shown in Figure 5, it was clearly evident that in treated cells, GAPDH was mainly located in the cytoplasm with a higher expression. Sirt1 was found in the cytoplasm of treated cells, while in untreated control samples Sirt1 was found mainly in the nucleus (Figure 5).

#### 3.1.4. Alizarin Red Assay

**Figure 6:**
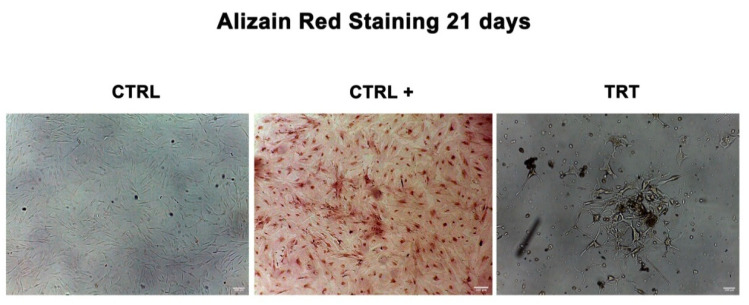
Differentiation of treated WJ-MSCs after 21 days. Differentiation of treated cells (TRT) as compared to untreated control cells, cultured in basic growing medium. Positive controls (CTRL +) were cells cultured in the presence of osteogenic differentiation medium. Scale bar = 100 µm. An average was generated from three technical replicates.

After 21 days of differentiation, the morphology of treated WJ-MSCs (TRT) was evaluated by light microscopy (Leica, Nussloch, Germany). Figure 6 shows that TRT cells cultured in the presence of the osteogenic conditioned medium did not acquire the typical morphology of mature osteocytes, as compared to positive control cells (CTRL+), in which intracellular calcium deposition was evident.

#### 3.1.5. Optical Microscope Analysis

**Figure 7:**
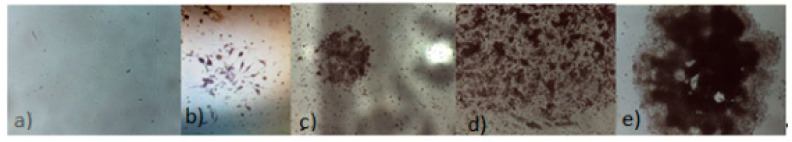
Sequential optical microscopy analysis of WJ-MSCs: (**a**) before treatment; (**b**) after 5 days of treatment; (**c**) 7 days of treatment; (**d**) 8 days of treatment; € 10 days of treatment. Scale bar = 40 µm.

We observed progressive phenotypical changes after 10 days in culture when stem cells were cultured with HepG2-exhausted medium (Figure 7).

## 4. Discussion

WJ-MSCs are becoming an interesting tool for allogenic transplantation in different kinds of disease, and also for COVID-19 complications [6,7,8]. Therefore, understanding the behavior of MSCs within the recipient tissues could be of great interest in assessing the safety of this kind of procedure. As reported by other authors, progenitor cells’ microenvironments could influence their potential malignant transformation [40]. Here, we reveal important results on the potential influence of HepG2 hepatocarcinoma cells on WJ-MSCs’ features and malignant transformation.

### 4.1. c-Myc’s Power

Other authors found that the overexpression of *c-Myc* increases the expression of *SIRT1*. *c-Myc* is able to induce *SIRT1* transcription by binding to its promoter [52,53,54,55,56,57]. *SIRT1* is a Class III histone deacetylase (*HDAC*) involved in gene regulation, maintaining genome stability, apoptosis, autophagy, senescence, aging, and tumorigenesis [52,53,54,55,56,57]. Several authors have highlighted a dual role of *SIRT1* as both a tumor suppressor and a tumor promoter [52,53,54,55,56,57]. *SIRT1* contributes to cell survival through inhibition of GAPDH translocation in the nucleus [58]. GAPDH is an enzyme of the glycolytic pathway, acting in the cytoplasm. It lacks any nuclear localization signal; nevertheless, it translocates to the nucleus via its interaction with the E3 ubiquitin ligase Siah [59]. SIRT1 prevents the nuclear translocation of GAPDH in response to apoptotic stress via a direct interaction [59]. The nuclear translocation of GAPDH is important for the cell signaling that activates apoptosis [59,60]. Moreover, SIRT1 inhibits the expression of *p53*-regulated genes, such as *p21* (*WAF1/CIP1*) [52,53,54,55,56,57,58,59,60], by inducing *p53* deacetylation, thus preventing cell senescence and apoptosis [52,53,54,55,56,57,58,59,60]. *p21* is a cell-cycle inhibitor that promotes both CDK/cyclin inhibition and cell-cycle arrest during the G1/S phase [61,62]. Our results showed a path in which WJ-MSCs exposed to medium exhausted by HepG2 cells exhibited an increase in the expression levels of *c-Myc*, *SIRT1*, and *GAPDH*, and a simultaneous decrease in *p53* and *p21* gene expression (Figure 2). These results confirmed that the overexpression of *SIRT1* is able to inhibit the *p53/p21*cip pathway and some related events, such as cell differentiation [61,62]. Here, we highlight that *p21* downregulation in treated WJ-MSCs (as compared to untreated controls) is related to an increase in cell proliferation, further implied by the greater expression of the stemness gene *c-Myc*, able itself to inhibit *p21* [62,63].

We also observed a downregulation of the expression of the epigenetic regulating gene *DNMT1* (Figure 1). On the other hand, no significant differences in *SOX2* or *NANOG* gene expression were observed between control and treated WJ-MSCs (Figure 1). These results revealed for the first time a pathway involving the capability of *c-Myc* to potentiate SIRT1 action, which in turns blocks GAPDH in the cytoplasm (Figure 5) [52,53,54,55,56,57,58]. Furthermore, SIRT1 potentiates the expression of *Oct-4* [22,63] (Figure 1), one of the crucial pluripotency-regulating factors in mesenchymal and embryonic stem cells [22,63].

Considering previous findings described by other authors [52,53,54,55,56,57,58], we hypothesize that the downregulation of *DNMT1* observed in this study (Figure 1) could induce the overexpression of *Oct-4* and *c-Myc*, which, in turn, could enhance *SIRT1* gene expression. This event may be responsible for blocking GAPDH in the cytoplasm (as is clearly shown in Figure 5). As shown by confocal microscopy analysis (TCS SP5, Leica) higher concentrations of SIRT1 and GAPDH were located in the cytoplasm of treated cells as compared to untreated control cells, where SIRT1 and GAPDH can be observed mainly in the nucleus. Furthermore, in treated cells, an increased cell proliferation was observed; this could be related, as already known, to a greater availability of the glycolytic pathway, and to SIRT1-mediated downregulation of *p53/p21* gene expression (Figure 2), thus counteracting stem-cell senescence [52,53,54,55,56,57,58]. These events were further supported by the upregulation of stemness-related genes such as *c-Myc* (Figure 1). Our results also showed that treated cells did not undergo differentiation toward the mesenchymal phenotype when stimulated with the conditioned differentiation medium (Figure 7), indicating an attempt by the cell to maintain a prolonged “childhood” condition. SIRT1 activation by c-Myc, together with p53 deacetylation, provides a mechanism able to promote c-Myc-induced cell proliferation by suppressing apoptosis and senescence.

### 4.2. MicroRNA’s Power

In the present paper, we also analyzed the expression levels of miR-185, miR-145, and miR-148, three miRNAs able to interfere with the expression of *c-Myc, Oct-4*, and *DNMT1*, which were also evaluated in our study.

miR-145 may facilitate differentiation by repressing the core pluripotency factors Oct-4 and SOX2, thus silencing the self-renewal program [40,41,42,49]. The loss of miR-145 expression in most human tumors and cancer cell lines may provide a favorable environment for cell survival [40,41,42]. Our results showed that miR-145-containing exosomes could be found in the culturing medium of WJ-MSCs, while progressively disappearing after cell exposure to HepG2-exausted medium. All the miRNAs analyzed in our study were mainly retained inside the cells (Figure 3 and Figure 4), being able to regulate stemness genes, as it was demonstrated by us in previous studies [40,41,42,49].

**Figure 8:**
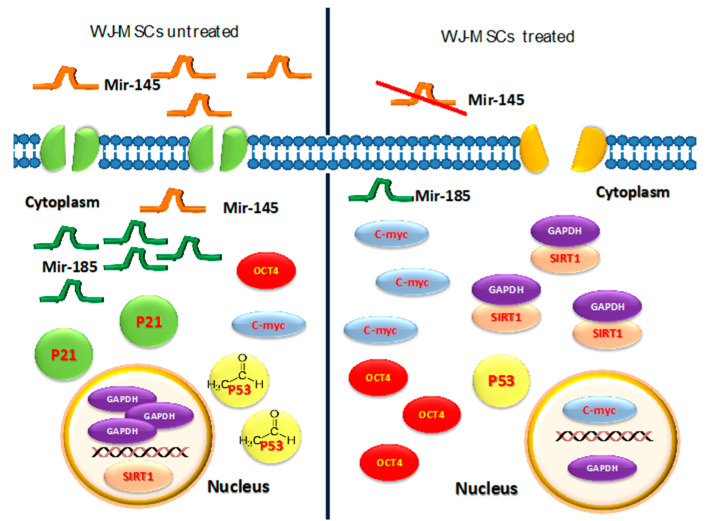
Differences in gene expression patterns between Treated/Untreated WJ-MSCs.

In particular, Figure 3 and Figure 4 show a progressive decrease of these miRNAs in treated cells, along with a progressive loss of their ability to undergo differentiation toward the mesenchymal phenotype (Figure 6), resembling the maintenance of a “childhood-like” signature. Nevertheless, the upregulation of miR-185 can lead to cell-cycle arrest [40,41,42], stopping cell proliferation and leading to a decreased *c-Myc* expression. Our results demonstrated a downregulation of miR-185 after treatment with the HepG2-exhausted medium (Figure 3) confirming the hypothesis of a permissive environment able to maintain a multipotent and a proliferative phenotype (Figure 8). Our results seem to indicate that a condition required for cell transformation into a proliferative phenotype is the impairment of cell commitment and differentiation, and the establishment of a condition of prolonged stemness beyond the established biological time. We hypothesize that an unfavorable biological environment may contribute to the dysregulation of normal biological cell homeostasis, as observed by us. Here, we clearly show that in treated WJ-MSC cultures, some cells seem to present a highly proliferative phenotype, resembling the behavior of HepG2 cells (Figure 8).

Following these considerations, we may assume the establishment of an autocrine/paracrine circuitry first triggered by HepG2-exhausted medium, and then stably maintained.

## 5. Conclusions

We observed the behavior of stem cells exposed to stimuli deriving from a tumor cell line, namely HepG2 cells. A greater expression of the stemness-related genes, together with a decrease of the miRNAs regulating these genes, was clearly evident. We also observed a related increase in cell proliferation, a stop of differentiation, and a maintenance of stem cell potency. Our aim was to understand the journey of stem cells towards the maintenance of stemness, which could lead to a dangerous proliferative phenotype. Further studies are needed in order to understand if our experiments could be translated in future clinical applications, for example by introducing plasma cleaning after tumor removal.

## Figures and Tables

**Table 1:** Primer sequences.

Primer Name	Forward	Reverse
***P16***	CAACGCACCGCCTAGTTACGG	AACTTCGTCCTCCAGAGTCGC
***P19***	GCCTTCGGCTGACTGGCTGG	TCGTCCTCCAGAGTCGCCCG
***P21***	CAAAGGCCCGCTCTACATCTT	AGGAACCTCCATTCACCCGA
***P53***	TGGCCTTGAAACCACCTTTT	AACTACCAACCCACCAGCCAA
***Oct-4***	GAGGAGTCCCAGGACATCAA	CATCGGCCTGTGTATATCCC
***SOX2***	CCGTTCATGTAGGTCTGCGAGCTG	CAACGGCAGCTACAGCATGATGC
***NANOG***	CATGAGTGTGGATCCAGCT	CCTGAATAAGCAGATCCAT
***c-Myc***	GGACGACGAGACCTTCATCAA	GCACCGAGTCGTAGTCGAG
***GAPDH***	GAGTCAACGGATTTGGTCGTGA	CTCCTTGGGCCGCGCATCAT
***DNMT1***	CGTCCGAGCGTCACACA	GAGCCTTTGCCATTCTTCGC
***SIRT1***	CATTTCCATGGCGCTGAGG	TGCTGGTGGAACAATTCCTGT

**Table 2:** miRNA collection: accession number, symbol, sequence, and identification number used in this study of miRNA.

Accession ID Number	Symbol	Sequence
MIMAT0000437	hsa-miR-145-5p	GUCCAGUUUUCCCAGGAAUCCCU
MIMAT0000243	hsa-miR-148a-3p	UCAGUGCACUACAGAACUUUGU
MIMAT0004611	hsa-miR-185-3p	AGGGGCUGGCUUUCCUCUGGUC
